# TRPV Subfamily (TRPV2, TRPV3, TRPV4, TRPV5, and TRPV6) Gene and Protein Expression in Patients with Ulcerative Colitis

**DOI:** 10.1155/2020/2906845

**Published:** 2020-05-08

**Authors:** Joel J. Toledo Mauriño, Gabriela Fonseca-Camarillo, Janette Furuzawa-Carballeda, Rafael Barreto-Zuñiga, Braulio Martínez Benítez, Julio Granados, Jesus K. Yamamoto-Furusho

**Affiliations:** ^1^Inflammatory Bowel Disease Clinic. Department of Gastroenterology, Instituto Nacional de Ciencias Médicas y Nutrición Salvador Zubirán, Mexico City, Mexico; ^2^MD/PhD Program (PECEM), Facultad de Medicina, Universidad Nacional Autónoma de México, Av. Ciudad Universitaria 3000, C.P. 04360 Coyoacán, México City, Mexico; ^3^Department of Immunology and Rheumatology, Instituto Nacional de Ciencias Médicas y Nutrición Salvador Zubirán, Mexico City, Mexico; ^4^Department of Endoscopy, Instituto Nacional de Ciencias Médicas y Nutrición Salvador Zubirán, Mexico City, Mexico; ^5^Department of Pathology, Instituto Nacional de Ciencias Médicas y Nutrición Salvador Zubirán, Mexico City, Mexico; ^6^Department of Transplantation, Instituto Nacional de Ciencias Médicas y Nutrición Salvador Zubirán, Mexico City, Mexico

## Abstract

**Introduction:**

TRPVs are a group of receptors with a channel activity predominantly permeable to Ca^2+^. This subfamily is involved in the development of gastrointestinal diseases such as ulcerative colitis (UC). The aim of the study was to characterize the gene and protein expression of the TRPV subfamily in UC patients and controls.

**Methods:**

We determined by quantitative PCR the gene expression of TRPV2, TRPV3, TRPV4, TRPV5, and TRPV6 in 45 UC patients (29 active UC and 16 remission UC) and 26 noninflamed controls. Protein expression was evaluated in 5 *μ*m thick sections of formalin-fixed, paraffin-embedded tissue from 5 customized severe active UC patients and 5 control surgical specimens.

**Results:**

TRPV2 gene expression was increased in the control group compared with active UC and remission patients (*P* = 0.002 and *P* = 0.05, respectively). TRPV3 gene expression was significantly higher in controls than in active UC patients (*P* = 0.002). The gene expression of TRPV4 was significantly higher in colonic tissue from patients with remission UC compared with active UC patients (*P* = 0.05) and controls (*P* = 0.005). TRPV5 had significantly higher mRNA levels in a control group compared with active UC patients (*P* = 0.02). The gene expression of TRPV6 was significantly higher in the colonic tissue from patients with active UC compared with the control group (*P* = 0.05). The protein expression of TRPV2 was upregulated in the mucosa and submucosa from the controls compared with the UC patients (*P* ≤ 0.003). The protein expression of TRPV3 and TRPV4 was upregulated in all intestinal layers from the controls compared with the UC patients (*P* < 0.001). TRPV5 was upregulated in the submucosa and serosa from the controls *vs.* UC patients (*P* < 0.001). TRPV6 was upregulated in all intestinal layers from the UC patients vs. controls (*P* ≤ 0.001).

**Conclusion:**

The TRPV subfamily clearly showed a differential expression in the UC patients compared with the controls, suggesting their role in the pathophysiology of UC.

## 1. Introduction

Inflammatory bowel disease (IBD) is a group of diseases that comprises Crohn's disease (CD) and ulcerative colitis (UC); both disorders are characterized by disturbances in the immune system and abnormal function of the gastrointestinal tract [1, 2]. Neuronal inflammatory pathways have also been described to be an important mechanism that participates in the development of IBD [3].

Transient receptor potential channels (TRP) constitute a distinct superfamily of ion channels and are distantly related to voltage-gated K^+^, Na^+^, and Ca^2+^ superfamilies. Thus, transient receptor potential channels of the vanilloid subtype (TRPV) subfamily comprise channels critically involved in nociception and thermosensitivity (TRPV1-4), whereas TRPV5 and TRPV6 are involved mainly in Ca^2+^ absorption/reabsorption [4].

TRPV2 is activated by noxious heat (>53°), mechanic stimulus (stretching and swelling), IGF-1, HA, 2-APB, cannabidiol, and probenecid [5, 6].

In a model of TRPV2-deficient mice, the severity of DSS-induced colitis was lower in macroscopic, microscopic, and immunohistochemical levels in comparison with wild-type animals [7]. These findings could be an effect from a reduced recruitment of macrophages to inflamed tissue, but it has been also suggested that they could rely on the fact that TRPV2 participates in the regulation of the number and function of Th and Tc cells [8].

TRPV3 is a channel that is highly sensitive to camphor, carvacrol, menthol, eugenol, caravel, turmoil, and by warm temperatures close to the core body temperature [9].

TRPV4 is a polymodal gated TRP channel that is activated by a diverse range of stimuli, including acidic pH, temperature, mechanical stress, the synthetic 4*α*-PPD, and arachidonic acid metabolites (epoxyeicosatrienoic acids) [10]. Fichna et al. induced colitis in mice by the intracolonic administration of TNBS, and they observed that mice treated with the TRPV4 antagonist RN1734 showed a significant protection for the development of signs of colitis [11], suggesting that TRPV4 could constitute a promising pharmacological target for IBD treatment.

The TRPV5 and TRPV6 are highly Ca^2+^-sensitive channels. They are responsible for limiting the rate of Ca^2+^ entry into cells during transcellular Ca^2+^ reabsorption [12–14]. The functional roles of TRPV5 and TRPV6 are interconnected with each other, given that TRPV5 knockout mice upregulate their intestinal TRPV6 expression to compensate for the negative Ca^2+^ balance caused by the loss of TRPV5-mediated Ca^2+^ reabsorption in the kidney [15–17]. Changes in the expression of TRPV5 and TRPV6 could be involved in the development of IBD and bone-related extraintestinal manifestations [18].

Previously, we published a study about gene and protein expression of TRPV1 in patients with UC, which established its association with the presence of intestinal inflammation [19].

Apart from the aforementioned data, the expression and distribution of these channels in the intestine from IBD patients are still insufficiently known. For this reason, the aim of the present study was to characterize gene and protein expression of the TRPV2-TRPV6 subfamily in patients with UC.

## 2. Materials and Methods

### 2.1. Patients

We determined the gene expression of TRPV2, TRPV3, TRPV4, TRPV5, and TRPV6 in a total of 71 patients and controls belonging to the Inflammatory Bowel Disease Clinic at the Instituto Nacional de Ciencias Médicas y Nutrición. Patients were diagnosed based on the established clinical, endoscopic, and histopathological criteria for UC and noninflammatory control subjects who underwent colonoscopy having a diagnosis of anemia under study, weight loss, or who underwent endoscopy. Individuals were divided into three groups: 29 active UC patients, 16 remission UC patients, and 26 controls.

Colon tissue specimens were evaluated by an independent GI pathologist and classified IBD histologically as being either active or inactive. Active disease was defined histologically by the presence of neutrophilic inflammation, including cryptitis and crypt abscesses. The mucosa that was uninvolved was defined as mucosa free of endoscopically and histologically active or chronic inflammation. In the case of inactive disease, chronic inflammation, crypt distortion, and/or lymphoid aggregates were common, although neutrophilic inflammation was absent.

Colonoscopy was performed for the calculus of the Mayo Score Activity Index. Disease extension was defined by colonoscopy. The disease activity was determined by Mayo Score and Riley criteria for endoscopic and histological activity, respectively, as well as a novel integral disease activity index for UC [20].

### 2.2. Tissue Samples

#### 2.2.1. Sample Processing and Gene Expression Analysis

The methodology used was based on a previous study that evaluated TRPV1 role in UC patients [19]. Acquisition of colonic biopsies from noninflammatory control patients and patients affected by UC was by a punch of the colonic mucosa. Only one biopsy was obtained from each patient, and then, it was preserved in cryovial tubes with 0.5 ml of nucleic acid preserver (RNAlater ®); then, they were kept on -70°C until the moment of RNA extraction.

RNA was extracted (according to manufacturer's methodology) from colonic mucosa biopsies using an RNA extraction kit (Roche® High Pure RNA Tissue Kit). Homogenization of biopsies was done with a lysis buffer (1 min) and then washed using 100% ethanol. Purification was done by columns, and the mix was centrifuged (13.000 × g, 15 s). Finally, 50 *μ*M of elution buffer was added to dilute total RNA.

Two hundred nanograms of total RNA was reverse transcribed into cDNA with the Transcriptor First Strand cDNA Synthesis Kit (Roche Diagnostics®, Mannheim, Germany).

Real-time PCR (RT-PCR) was performed using cDNA that resulted from retrotranscription as a substrate using LightCycler® 480 Probes Master kit and LightCycler® 480 Multiwell Plate 96. For the amplification of the regions of interest, the reaction was made in 10 *μ*l. Amplification was performed under the next conditions: a denaturation program (95°C, 10 min), 45 amplification cycles (denaturation (95°C, 10s), annealing (60°C, 10 s), extension (40°C, 30 s), and one cooling cycle (40°C, 30 s).

For the determination of gene expression (TRPV2, TRPV3, TRPV4, TRPV5, and TRPV6) and GAPDH (glyceraldehyde phosphate dehydrogenase as a constitutive gene), Light Cycler 480 (Roche Diagnostics®, Mannheim, Germany) thermocycler was used with quantitative validated assays (reproducibility and linearity), employing primers from the Universal ProbeLibrary SET (Human of Roche ®) and TaqMan probes for each gene as shown in Table 1.

#### 2.2.2. Immunohistochemistry

Immunohistochemical conditions (titration of primary and secondary antibodies) were standardized like in previous reports [19, 21, 22]. The TRPV2, TRPV3, TRPV4, TRPV5, and TRPV6 protein expression was determined on a single run, using 5 *μ*m thick sections of formalin-fixed paraffin-embedded tissue from 5 colectomized active UC patients and 5 controls with colon cancer patients (noninflamed zones were used as control tissue).

First, deparaffinization and rehydration was developed using xylene and graded alcohols. Antigen retrieval with citrate buffer and pH 6.0 during 10 min at 100°C was performed. Slides were washed with deionized water and then with a wash buffer. Tissues were treated with 3% H_2_O_2_ (ABC Staining System) and 10% of normal donkey serum (Santa Cruz Biotechnology), in order to block endogenous peroxidase and prevent binding of nonspecific proteins. Slides were incubated with the addition of rabbit anti-human (TRPV2, TRPV5), mouse anti-human (TRPV6), or goat anti-human (TRPV3, TRPV4) (Santa Cruz Biotechnology) diluted at 10 *μ*g/ml for 18 h at 4°C. Then, tissue specimens were incubated (60 min, room temperature) with goat anti-rabbit IgG antibody alkaline phosphatase conjugate, goat anti-mouse IgG antibody peroxidase conjugate, or donkey anti-goat IgG antibody peroxidase conjugate (Enzo Life Sciences, Inc., Farmingdale, NY, USA, and Santa Cruz Biotechnology) for detection of binding. Slides were incubated with the substrate permanent red (Sigma-Aldrich Co.) for alkaline phosphatase or diaminobencidine for peroxidase, during 10 min at room temperature. Sections were counterstained with Mayer's hematoxylin (Lillie's modification) (Dako, Glostrup, Denmark) and immersed into a bath of ammonia water (7 mM/l), rinsed in deionized water (2-5 min), dehydrated, and mounted employing Faramount aqueous mounting medium (Dako). Normal human serum (1 : 100) was used instead of primary antibody in the case of negative control staining and the IHC universal negative control reagent (specific for rabbit, mouse, and goat antibodies) (Enzo Life Sciences). For reactive blank phosphate buffer saline-egg albumin (Sigma-Aldrich) was used instead of primary antibody. Nonspecific staining of endogenous enzyme block was excluded in case of controls.

### 2.3. Statistical Analysis

Gene expression statistical analysis was performed using Dunn's test of multiple comparisons, and overlays were deleted. Results are reported as mean ± SEM. A *P* value < 0.05 was considered as significant.

Protein expression was evaluated by a morphometric evaluation of immune-stained sections in a blinded manner. TRPV2, TRPV3, TRPV4, TRPV5, and TRPV6 immunopositive cells were counted in three fields at 320x and were reported as the percentage of immunoreactive cells of the inflammatory infiltrates located at the mucosa, submucosa, muscular layer, and serosa. Mean ± SEM was reported for each case. Software used to be Image-Pro Plus v.5. Statistical analysis of continuous variables was performed by *t*-test and the nonparametric Mann–Whitney rank-sum test.

## 3. Results

A total of 45 patients with UC (19 men and 26 women; mean age: 43.5 years) and 26 controls (8 men and 18 women; mean age: 54.0 years) were evaluated. With regard to disease activity, 29 had active disease and 16 were in remission according to Yamamoto-Furusho score. The extent of the disease was evaluated by using total colonoscopy. Biopsies were taken from different segments of colon in all cases. The Montreal classification was used to define the extent of UC: 19 had pancolitis (E3), 3 had left-sided colitis (E2), 9 had proctitis (E1), and 2 were not classifiable.

With regard to the medical treatment, 35 patients were taking sulfasalazine or 5-aminosalicylates (5-ASA), 10 were taking oral or systemic steroids, 6 were taking azathioprine, and 1 was treated with anti-TNF therapy. Sociodemographic and clinical characteristics are shown in Table 2.

### 3.1. Differential Gene Expression of the TRPV Subfamily in Patients with Ulcerative Colitis

**Figure 1:**
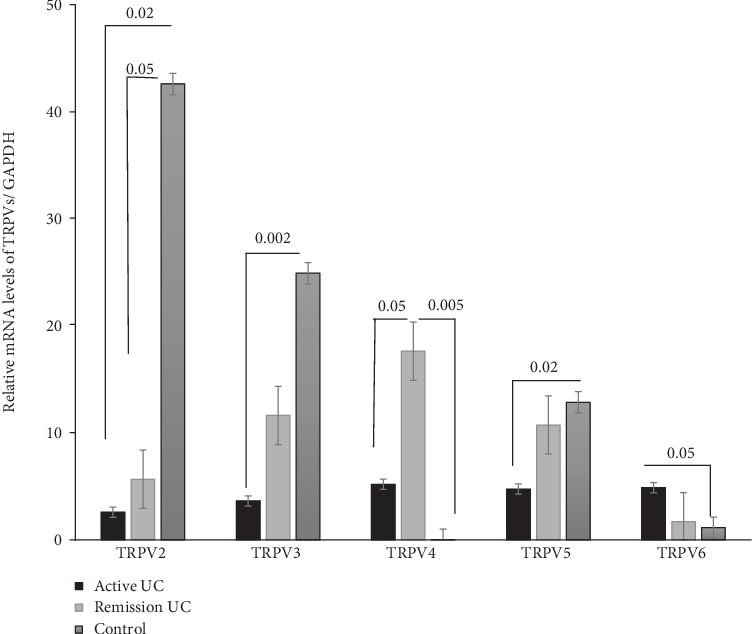
TRPV2, TRPV3, TRPV4, TRPV5, and TRPV6 gene expression quantified by RT-PCR in colonic mucosa from patients with active and remission UC compared to non-IBD controls. Bars show mean ± standard error of the mean of transcript levels from UC patients with GAPDH as constitutive gene determined by 2^-*ΔΔ*Ct^. ^∗^*P* value < 0.05 was considered as significant.

TRPV2 gene expression was increased in the control group compared with active and remission UC patients (*P* = 0.002 and *P* = 0.05). No statistically significant difference was found among patients with active UC compared with remission patients. In the same vein, TRPV3 gene expression was significantly higher in the controls than in the active UC patients (*P* = 0.002). The gene expression of TRPV4 was significantly higher in the colonic tissue from patients with remission UC compared with the active UC patients (*P* = 0.05) and controls (*P* = 0.005). No statistically significant difference was found among patients with active UC compared with the control group. TRPV5 has significantly higher mRNA levels in the control group compared with the active UC patients (*P* = 0.02). No statistically significant differences were found between patients with remission UC compared with the active UC and control groups. Finally, TRPV6 gene expression was significantly higher in active UC patients compared with the controls (*P* = 0.05) (Figure 1).

### 3.2. Protein Expression of TRPV2, TRPV3, TRPV4, TRPV5, and TRPV6 in Patients with UC

Histological findings of UC included expansion of chronic inflammation (lymphoplasmacytic infiltrates) in the mucosa, cryptitis that in some cases progressed to crypt abscesses, and mucosal ulceration. A shortening of the crypts and distortion of their branches, as well as irregular luminal border, were observed. A reduction of the intraepithelial mucin was also detected. In a mere morphological analysis, it was observed that mononuclear inflammatory infiltrates, enriched in lymphocytes and plasma cells, were also present in the lamina propria. In most of the cases, submucosal fibrosis was present.

**Figure 2:**
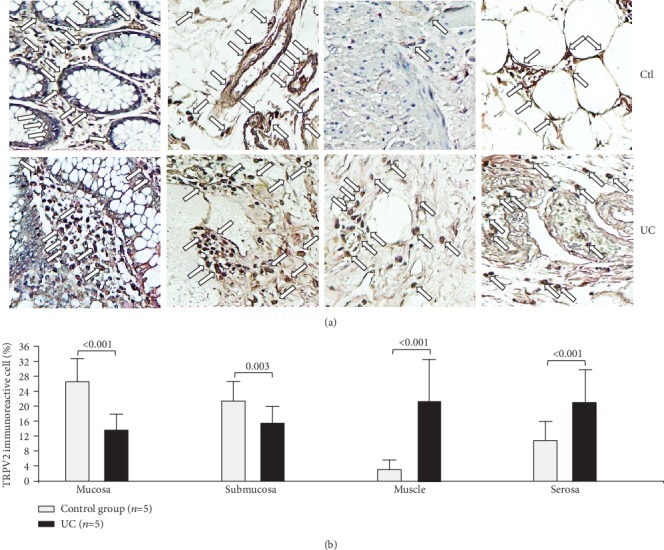
Protein expression of TRPV2 in intestinal tissue from patients with UC and controls. TRPV2 protein expression in colonic tissue samples obtained from patients with severe ulcerative colitis and noninflamed colonic tissue. (a) Immunoperoxidase photomicrographs of ulcerative colitis (lower panel, *n* = 5) and non-IBD colonic tissue (control; upper panel, *n* = 5). Arrows indicate TRPV2 immunoreactive cells in the mucosa, submucosa muscular, and serosa layers. Original magnification was ×320. (b) TRPV2-producing cell percentage in noninflamed colonic tissues (control, *n* = 5) and active UC patients (*n* = 5) is shown in bars.

The protein expression of TRPV2 was higher in the mucosa (*P* < 0.001) and submucosa (*P* = 0.003), but lower in the muscular (*P* < 0.001) and cells from the serosa (*P* < 0.001) in the case of the controls in comparison to patients affected by UC (Figure 2).

**Figure 3:**
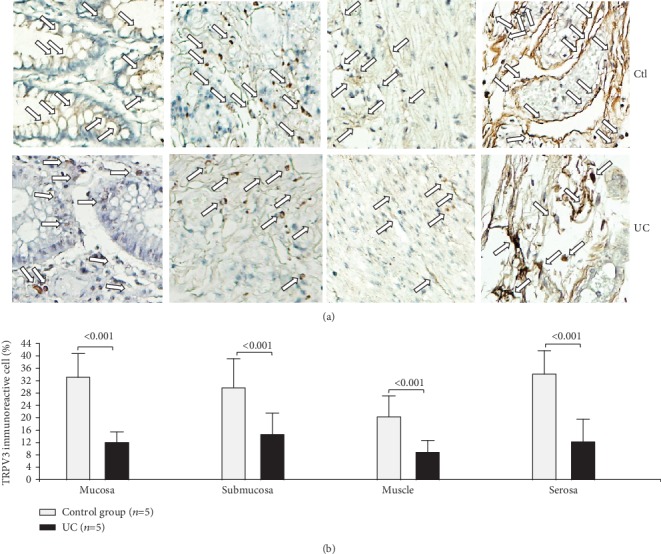
Protein expression of TRPV3 in intestinal tissue from patients with UC and controls. TRPV3 protein expression in slides with colonic tissue obtained from patients with severe ulcerative colitis and noninflamed colonic tissue. (a) Immunoperoxidase photomicrographs of ulcerative colitis (lower panel, *n* = 5) and non-IBD colonic tissue (control; upper panel, *n* = 5). Arrows indicate TRPV3 immunoreactive cells in the mucosa, submucosa muscular, and serosa layers. Original magnification was ×320. (b) TRPV3-producing cell percentage in noninflamed colonic tissues (control, *n* = 5) and active UC patients (*n* = 5) is shown in bars.

Detection *in situ* of TRPV3 was higher in the case of the controls in comparison to patients affected by UC across all intestinal layers (mucosa, *P* < 0.001; submucosa, *P* < 0.001; muscular, *P* < 0.001; and serosa, *P* < 0.001; Figure 3).

**Figure 4:**
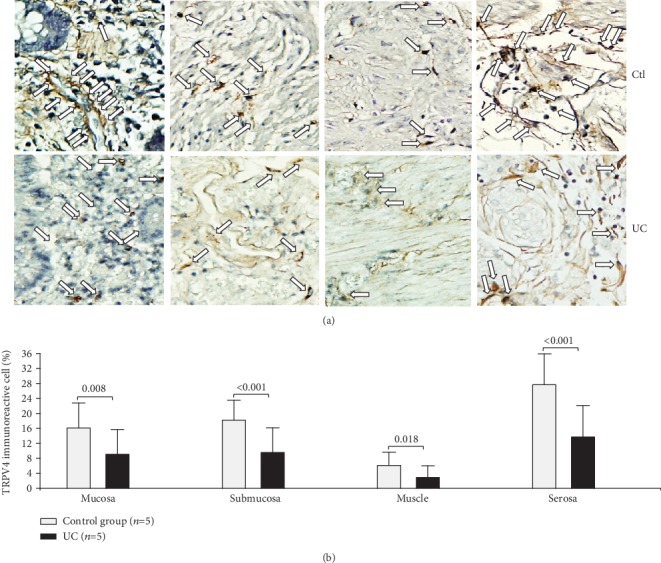
Protein expression of TRPV4 in intestinal tissue from patients with UC and controls. TRPV4 protein expression in slides with colonic tissue obtained from patients with severe ulcerative colitis and noninflamed colonic tissue. (a) Immunoperoxidase photomicrographs of ulcerative colitis (lower panel, *n* = 5) and non-IBD colonic tissue (control; upper panel, *n* = 5). Arrows indicate TRPV4 immunoreactive cells in the mucosa, submucosa muscular, and serosa layers. Original magnification was ×320. (b) TRPV4-producing cell percentage in noninflamed colonic tissues (control, *n* = 5) and active UC patients (*n* = 5) is shown in bars.

TRPV4 protein detection was lower in the mucosa (*P* = 0.008), submucosa (*P* < 0.001), muscular(*P* = 0.018), and serosa (*P* < 0.001) of UC patients compared to the control group (Figure 4).

**Figure 5:**
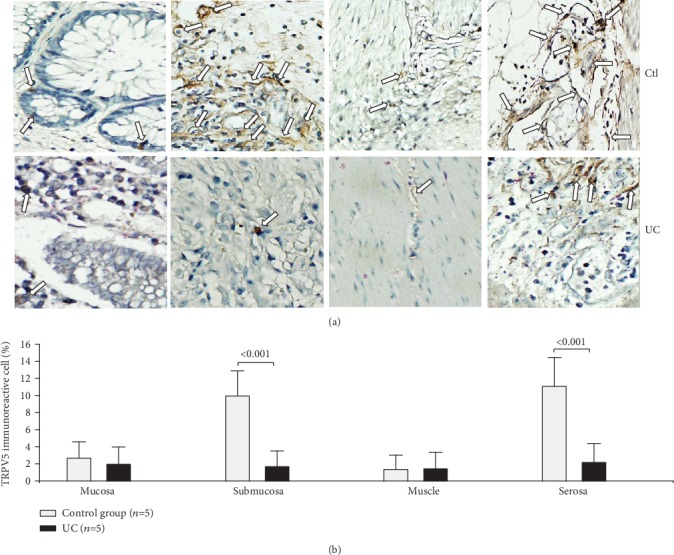
Protein expression of TRPV5 in intestinal tissue from patients with UC and controls. TRPV5 protein expression in slides with colonic tissue obtained from patients with severe ulcerative colitis and noninflamed colonic tissue. (a) Immunoperoxidase photomicrographs of ulcerative colitis (lower panel, *n* = 5) and non-IBD colonic tissue (control; upper panel, *n* = 5). Arrows indicate TRPV5 immunoreactive cells in the mucosa, submucosa muscular, and serosa layers. Original magnification was ×320. (b) TRPV5-producing cell percentage in noninflamed colonic tissues (control, *n* = 5) and active UC patients (*n* = 5) is shown in bars.

The protein expression of TRPV5 appeared to be reduced across all intestinal layers although it was only significant in the submucosa (*P* < 0.001) and serosa (*P* < 0.001) in the case of the UC group in comparison to the controls (Figure 5).

**Figure 6:**
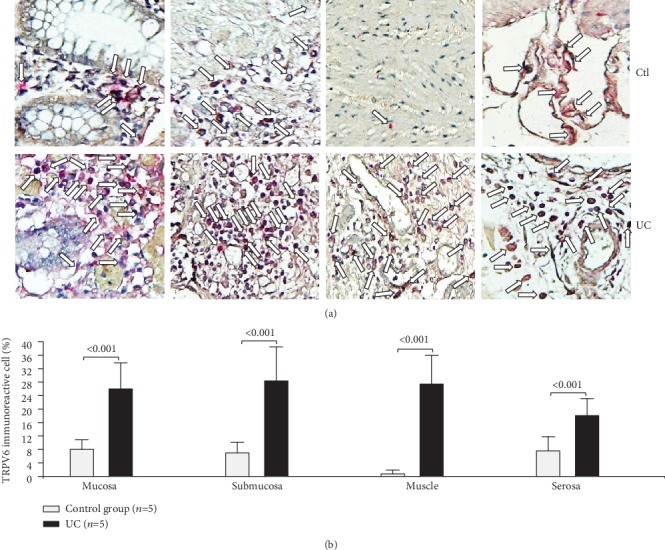
Protein expression of TRPV6 in intestinal tissue from patients with UC and controls. TRPV6 protein expression in slides with colonic tissue obtained from patients with severe ulcerative colitis and noninflamed colonic tissue. (a) Immunoperoxidase photomicrographs of ulcerative colitis (lower panel, *n* = 5) and non-IBD colonic tissue (control; upper panel, *n* = 5). Arrows indicate TRPV6 immunoreactive cells in the mucosa, submucosa muscular, and serosa layers. Original magnification was ×320. (b) TRPV6-producing cell percentage in noninflamed colonic tissues (control, *n* = 5) and active UC patients (*n* = 5) is shown in bars.

The TRPV6 protein expression was higher in every intestinal layer in the case of the UC group (mucosa, *P* < 0.001; submucosa, *P* < 0.001, muscular, *P* < 0.001; and serosa, *P* < 0.001; Figure 6).

## 4. Discussion

The TRPV subfamily has been involved in the pathophysiology of several gastrointestinal diseases such as gastroesophageal reflux disease (GERD), nonesophageal reflux disease (NERD), and irritable bowel syndrome (IBS) [17, 18].

Thus, in this study, we determined the gene and protein expression of the other members of the TRPV subfamily, in order to provide the characterization of these receptors in patients with active and remission UC.

Interestingly, the gene expression of TRPV2-5 was decreased in patients with active UC compared to the group of UC in remission and controls without intestinal inflammation.

Previously, we published a study about gene and protein expression of TRPV1 in which its association with the presence of intestinal inflammation was described [19]. Thus, in the present study, we determined the gene and protein expression of other members of the TRPV subfamily, in order to provide the characterization of these receptors in patients with active and remission UC [19].

In the case of TRPV2, the control group showed higher levels of TRPV2 gene expression compared to the active UC patients. While in the protein expression analysis, the internal layers of the intestine had an increased expression of TRPV2 in the mucosa (*P* < 0.001) and submucosa (*P* = 0.003) but lower in the muscular (*P* < 0.001) and serosa (*P* < 0.001) in the case of the controls in comparison to patients affected by UC. According to this result, a previous study also reported increased TRPV channel protein expression in mucosa epithelial cells of non-IBD control samples and UC patients [23]. Furthermore, in mice with DSS-induced colitis model, higher TRPV2 gene expression was associated with higher degrees of intestinal inflammation [7]. Regarding protein expression, such results could be related to a lower level of expression of TRPV2 by lymphoid cells, since the main cellular sources found in previous studies are T-cell lymphocytes in which the receptor shows a direct interaction with T-cell receptor and macrophages [23, 24]. TRPV2 activation has been associated with the production of IL-6 and IL-8, but such production is not documented in the case of the gastrointestinal tract [25].

Specific implications of TRPV3 in the gastrointestinal tract have not been explored. Nonetheless, this study has demonstrated that gene and protein TRPV3 downregulated expression was associated with disease activity, as previously reported by Rizopoulos et al. [23]. TRPV3 has already been proposed as a relevant mechanism for gastrointestinal inflammation, but there are still no studies about specific pathways [26].

Interestingly, the gene expression of TRPV4 in patients with UC in the remission and control groups was increased in comparison with active UC. The above information suggests that a high expression of TRPV4 could be related with a healthy colon state.

Conversely, in other studies, TRPV4 upregulation was associated with the presence of inflammation in UC patients and in colitis model mice [23, 27, 28], where the TRPV4 channel could increase the vascular permeability in colonic inflammation. TRPV4 might be involved in the production of IL-6 and IL-8 via ATP release for the development of inflammation [29].

In addition, the lack of TRPV5 appears to be correlated with the induction of UC. Radhakrishnan et al. [30] have demonstrated that TRPV5 channel expression at the renal tissue was involved in bone loss in experimental colitis. The plausible mechanism by which this TRPV5 channel could be involved in the intestinal inflammation is the activation of internalization of this channel via clathrin-dependent endocytosis to enter Ca2+ controlled by recycling cascades, but this mechanism has not yet been characterized in patients with UC [31].

It has been demonstrated that TRPV6 is particularly involved in the first step in calcium absorption in the intestine [32, 33]. This study has shown high gene and protein expression in all intestinal layers from UC patients, and it appears to be clearly related to disease activity.

Mechanistic studies required to reveal more clearly the implications of this subfamily in the pathophysiology of UC.

This transversal study is limited to Mexican population, and since diet is related to the function and expression of these channels, further studies in other populations with a bigger number of individuals are needed and also with a pertinent characterization of dietary patterns. Moreover, translational studies in UC are required since there is some evidence that TRPV subfamily members could play a role in the pathogenesis of intestinal inflammation in murine models, as well as human IBD.

## 5. Conclusion

The TRPV2-6 channels clearly showed a differential expression in UC patients compared to the controls, suggesting a possible role in the development of intestinal inflammation in UC patients. The description of TRPV 2-TRPV 6 expression is the first of its kind and may lay the groundwork for future investigations focused on the role of these channels in IBD.

## Figures and Tables

**Table 1:** Primers designs from Universal ProbeLibrary.

Gene	GeneBank	Oligonucleotides	Probe UPL
TRPV2	NM_016113.4	5′ ggtgttggcctgactgga 3′ 3′cagcccctgctactgagaa 5′	#3
TRPV3	NM_145068.2	5′aatctcgggctggttggt 3′ 3′ ccaacacgaaggcttctacttc 5′	#81
TRPV4^∗^	NM_021625.4 NM_147204.2 NM_001177428.1 NM_001177431.1 NM_001177433.1	5′ ccaggtaggcctcgatcc 3′ 3′ gctccttccagctgctctac 5′	#66
TRPV5	NM_019841.4	5′ tggggtctgttccagaattt 3′ 3′ ctgtccttcctggagcttgt 5′	#82
TRPV6	NM_018646.2	5′ gaaggagaggagactcccaga 3′ 3′ agagccgagatgagcagaac 5′	#84
GAPDH	NM_002046.3	5′ gcccaatacgaccaaatcc 3′ 3′agccacatcgctcagacac 5′	#60

^∗^Oligonucleotides were designed considering all the alternative splicing variations.

**Table 2:** Sociodemographic characterization of the patients and controls.

Clinical characteristics		Active UC (28)		Remission UC (16)		Controls (25)	
		*n*	%	*n*	%	*n*	%
Gender	Male	11	37.9	8	50	8	30.77
	Female	18	62.1	8	50	18	69.23
Age (median, range)		40	(24-72)	47	(16-75)	54	(23-74)
Age at diagnosis (mean, SD)		31	(6-56)	34	(9-66)		
Years of evolution (median, range)		6	(1–24)	7	(0-17)		

Extent of disease	E1	7	24.1	2	12.5		
	E2	2	6.8	1	6.3		
	E3	18	62.1	11	68.8		
	Not classifiable	2	6.9	2	12.5		

Extraintestinal manifestations	Present	10	34.5	9	56.3		
	Absent	17	58.6	5	31.3		
	Not documented	2	6.9	2	12.5		

Clinical course of disease	Initially active	11	37.9	2	12.5		
	Intermittent	1	48.2	9	56.3		
	Continuous	2	6.9	2	12.5		
	Not documented	2	6.9	3	18.8		

Medical treatment	5-ASA	26	89.7	9	56.3		
	Steroids	7	24.1	3	18.8		
	Thiopurines	4	13.8	2	12.5		
	Anti-TNF*α*	1	3.4				

## Data Availability

Data will be provided based on requirement.

## Abbreviations

*μ*m: Micrometer
2-APB: 2-Amino diphenyl borate
4*α*-PPD: Phorbol ester 4*α* phorbol 12,13-didecanoate
ANOVA: Analysis of variance
Ca^2+^: Calcium
CD: Crohn's disease
DSS: Dextran sulphate sodium
g: Gravity
GI: Gastrointestinal
H_2_O_2_: Hydrogen peroxide
HA: Heat activator
IBD: Inflammatory bowel disease
IGF-1: Insulin-like growth factor 1
IgG: Immunoglobulin G
IHC: Immunohistochemistry
K^+^: Potassium
min: Minute
ml: Milliliter
mRNA: Messenger ribonucleic acid
pH: Potential hydrogen
Na^+^: Sodium
RNA: Ribonucleic acid
RT-PCR: Real-time polymerase chain reaction
s: Second
SD: Standard deviation
Tc: T-cytotoxic
Th: T-helper
TNBS: Trinitro benzene sulphonic acid
TRPV: Transient receptor potential vanilloid
UC: Ulcerative colitis.
